# Risk of Death and Adverse Effects in Patients on Liothyronine: A Multisource Systematic Review and Meta-analysis

**DOI:** 10.1210/clinem/dgaf449

**Published:** 2025-08-08

**Authors:** Suhani Bahl, Peter N Taylor, Lakdasa D Premawardhana, Mike Stedman, Adrian Heald, Colin M Dayan, Onyebuchi E Okosieme

**Affiliations:** Thyroid Research Group, Systems Immunity Research Institute, Cardiff University School of Medicine, Cardiff, Wales CF14 4XN, UK; Thyroid Research Group, Systems Immunity Research Institute, Cardiff University School of Medicine, Cardiff, Wales CF14 4XN, UK; Thyroid Research Group, Systems Immunity Research Institute, Cardiff University School of Medicine, Cardiff, Wales CF14 4XN, UK; Res Consortium, Andover SP10 5RG, UK; The School of Medicine and Manchester Academic Health Sciences Centre, University of Manchester, Manchester M14 4PX, UK; Thyroid Research Group, Systems Immunity Research Institute, Cardiff University School of Medicine, Cardiff, Wales CF14 4XN, UK; Thyroid Research Group, Systems Immunity Research Institute, Cardiff University School of Medicine, Cardiff, Wales CF14 4XN, UK; Diabetes Department, Prince Charles Hospital, Cwm Taf Morgannwg University Health Board, Merthyr Tydfil, Wales CF47 9DT, UK

**Keywords:** hypothyroidism, liothyronine, levothyroxine, adverse events, cardiovascular, death

## Abstract

**Context:**

Although some patients with hypothyroidism prefer combination therapy with liothyronine (LT3) and levothyroxine (LT4), the safety of LT3 remains unresolved.

**Objective:**

We undertook a multisource systematic review and meta-analysis of LT3 safety.

**Data Sources:**

We searched PubMed for articles relating to death, adverse events (AEs), and cardiovascular outcomes in LT3 users. We also searched AEs data in the UK Yellow Card scheme and US Food and Drug Administration Adverse Reporting System (FAERS).

**Data Extraction:**

Data was extracted independently by 2 reviewers. Out of 1814 articles identified, 52 studies were selected, comprising 21 randomized controlled trials (RCTs), 4 cohort studies, and 27 case reports. Meta-analyses were conducted for adverse outcomes in RCTs and cohort studies of combination vs monotherapy.

**Data Synthesis:**

LT3-related AEs were only reported with unregulated LT3 use or pharmacy compounding errors. LT3 and LT4 showed similar adverse severity profiles in the Yellow Card scheme. Disproportionality analysis in the FAERS database showed no increased LT3 safety signals. A meta-analysis of RCTs (n = 2128) showed a similar AEs risk for combination vs monotherapy [relative risk (RR) 1.22, 95% confidence interval (CI) 0.66-2.25]. A cohort study meta-analysis (LT3 vs LT4-only users, n = 630 254) showed no increased risk of atrial fibrillation (RR 1.10, 95% CI 0.74-1.63), heart failure (RR 1.54, 95% CI 0.95-2.47), or strokes (RR 0.86, 95% CI 0.11-6.75), but reduced mortality risk was observed for LT3 (RR 0.70, 95% CI 0.62-0.78).

**Conclusion:**

Our findings are reassuring that regulated LT3 use is not associated with the risk of death or serious AEs. More studies are needed to supplement existing data.

Thyroid hormones have been used in the treatment of hypothyroidism for over a century (1). Hypothyroidism affects about 2% to 5% of the global population, with increasing prevalence in women and older adults (2). Untreated hypothyroidism is characterized by symptoms such as lethargy, memory problems, depression, and weight gain (3, 4). Furthermore, hypothyroidism carries an increased risk of cardiovascular disease, abnormal lipid metabolism, and neurocognitive problems (3, 4). Early therapy for hypothyroidism relied on desiccated thyroid extracts (DTE), which was eventually replaced by synthetic preparations in the 1960s (5). Levothyroxine (LT4), a synthetic form of T4, subsequently became the treatment of choice due to its biochemical stability and peripheral conversion to the active hormone, T3 (2, 5, 6). LT4 is now the third most prescribed medication in the UK and is likely to become even more widely used in the future (7).

Most patients with hypothyroidism respond well to LT4 and report improvement in well-being, with TSH returning to normal within weeks of initiating treatment. However, observational studies suggest that a proportion of patients on LT4 alone continue to display impairment of psychological well-being compared with controls of similar age and sex (8, 9). For some patients, combination treatment with LT4 and liothyronine (LT3) offers an alternative treatment approach (10). Although the superiority of combination therapy over LT4 alone remains unproven in randomized controlled trials (RCTs) (11-13), meta-analyses have shown that patients with hypothyroidism prefer combination therapy over LT4 monotherapy (14). However, due to the unproven efficacy data, major international guidelines continue to recommend LT4 as the standard of care, with combination therapy only reserved for patients who have not equivocally derived symptomatic benefit from LT4 monotherapy (15-18).

A potential hindrance to the use of LT3, however, is the perception that it has a less favorable safety profile than LT4 and that it exerts adverse effects on cardiovascular and bone health (19). This perceived risk of harm has contributed to a reluctance to prescribe LT3 and DTE among clinicians (8). These concerns do not, however, appear to be based on substantial evidence. Systematic reviews on LT3 safety are limited and have been confined to the analyses of short-term adverse outcomes in treatment efficacy trials (11, 12). A recent industry-funded review evaluated LT3 safety, including market safety information pertaining to an LT3-LT4 combination tablet (20). However, the review was nonsystematic, and the safety reports were limited to the authors' own branded product (20). A comprehensive evaluation of short- and long-term safety outcomes of LT3 using validated systematic review and pharmacovigilance methods is therefore lacking.

We recently reported on a case of sudden death in a patient who was suspected to have ingested self-procured LT3 (21). This case thus prompted us to undertake a systematic review on the risk of death and severe adverse reactions in patients taking LT3 using a variety of sources from the published literature and pharmacovigilance databases. Our aim was to systematically summarize the available safety data on LT3 in the treatment of hypothyroidism using a multisource approach.

## Methods

We conducted a systematic review of published case reports, cohort studies, RCTs, and reports in drug monitoring databases to determine the risk of death and major adverse reactions in patients on treatment with LT3.

### Search Criteria

We searched PubMed from database inception to October 2024, without language restrictions, using a combination of the search terms liothyronine, triiodothyronine, T3, desiccated thyroid extracts, combination therapy, AND death, mortality, fatality, major adverse reactions, major adverse events (AEs), cardiovascular disease, strokes, heart failure, atrial fibrillation, myocardial infarction, AND hypothyroidism, Hashimoto's thyroiditis, autoimmune thyroiditis. We selected relevant articles based on information from their titles and abstracts with full texts accessed if the abstract information was insufficient to exclude the study. Additional papers were obtained from within references cited in relevant articles or from adverse case reports cited in pharmacovigilance databases. We also searched online research databases, namely ClinicalTrials.gov (www.clinicaltrials.gov) and the UK Clinical Study registry (www.isrctn.com/). Two reviewers (S.B., O.O.) reviewed the abstracts, and discrepancies were resolved by consensus or referral to a third reviewer (C.M.D.).

### Study Inclusion

Studies were included in the review if they were (1) case reports or case series that reported on death or serious adverse reactions in patients on LT3; (2) observational studies that addressed the risk of death, cardiovascular outcomes, or severe AEs in LT3- vs LT4-treated patients; and (3) RCTs of combination therapy vs LT4 monotherapy that provided information on AEs.

### Pharmacovigilance Databases

#### The US Food and Drug Administration Adverse Reporting System database

Drug safety data were obtained from the US Food and Drug Administration Adverse Reporting System (FAERS) (22). FAERS is a publicly accessible database of drug adverse reports submitted by members of the public, healthcare professionals, and pharmaceutical companies (https://fis.fda.gov) (22). We ran a search in FAERS for all AEs from 1968 to 2024 that were reported in association with LT3 and LT4 comprising all the different preparations of these drugs (eg, levothyroxine sulphate, levothyroxine, sodium, etc.) including generic and brand names. Results were further searched for in-text references of published case reports pertaining to LT3 to supplement the case report literature search.

#### Yellow Card scheme

We also searched the UK Medicines and Healthcare Regulatory Agency (MHRA) Yellow Card scheme. The Yellow Card scheme is run by the UK MHRA, through which it collects and monitors information on suspected safety concerns involving medicines and healthcare products. Adverse event reports are held online in MHRA's publicly available Interactive Drug Analysis Profiles reports (https://yellowcard.mhra.gov.uk/idaps). In the Interactive Drug Analysis Profiles database, we searched for AEs reported in association with LT4 and LT3 for the period 1967 to 2024 (23). Reports were grouped by severity, and AEs were classified by the primary system organ class as defined by the Medical Dictionary for Regulatory Activities terminology (version 27.0) (24).

### Data Analysis

Case reports and case series were summarized according to patient demographics (age, sex), indication for liothyronine use, dose and duration of treatment, and AE outcome. For cohort studies, we assessed the methodological quality of studies using the Newcastle Ottawa Scale for the assessment of observational studies (25). Effect estimates are presented for individual studies as reported by the authors. Where feasible, a meta-analysis of cohort studies was undertaken with effect estimates summarized as risk ratios and 95% confidence intervals (CIs) using a random effects model with the restricted maximum likelihood ratio method. Heterogeneity was assessed using I2 statistics.

For RCTs of combination LT4/LT3 vs LT4, the occurrence of AEs was summarized as counts. For each study, we counted the numbers of participants in each study arm who discontinued medications or withdrew from the trial after recruitment due to the development of side effects or AEs that were judged by the trial authors to be related to the study drugs. These included participants who discontinued medications or withdrew from the trial after developing features of thyrotoxicosis, palpitations, cardiac arrhythmias, strokes, gastrointestinal side effects, headaches, etc. We did not count individuals who withdrew for personal or administrative reasons, protocol violation, or lack of treatment effect or following the development of unrelated diseases such as cancer. Studies that provided inadequate information to determine withdrawals in each study arm were excluded from the meta-analysis in contrast to studies that reported 0 withdrawals, which were included. Pooled relative risks were derived using a random effects model with the restricted maximum likelihood ratio method. Relative risks were calculated first for all eligible studies and then for studies without 0 counts. Adjustment for 0 counts was undertaken using a continuity correction method as described by Sweeting et al (26).

### Disproportionality Analysis

We used disproportionality analysis to evaluate potential associations between LT3 and LT4 and severe AEs, including fatal events. A disproportionality analysis is a validated pharmacovigilance tool for evaluating associations between drugs and AEs. This analysis was conducted in the FAERS database but was not feasible in the Yellow Card scheme or other pharmacovigilance databases, as the total number of all reactions to all drugs was not readily accessible as in FAERS. For each drug, we derived reporting odds ratios (RORs) and proportional reporting ratios. These ratios are based on the observed counts of AEs reported for a drug with respect to the expected count based on all other drugs in the database (27, 28). The ratios were calculated from 2 × 2 contingency tables as shown in Supplementary Table S1 (29). Signals were considered signiﬁcant for both measures if the ratios were >1.0 with 95% CI >1.0. In confirmatory analyses, we calculated the information component (IC) with 95% CI (Supplementary Table S1) (29). This was considered significant if the IC was >0.

## Results

### Study Selection

**Figure 1.**
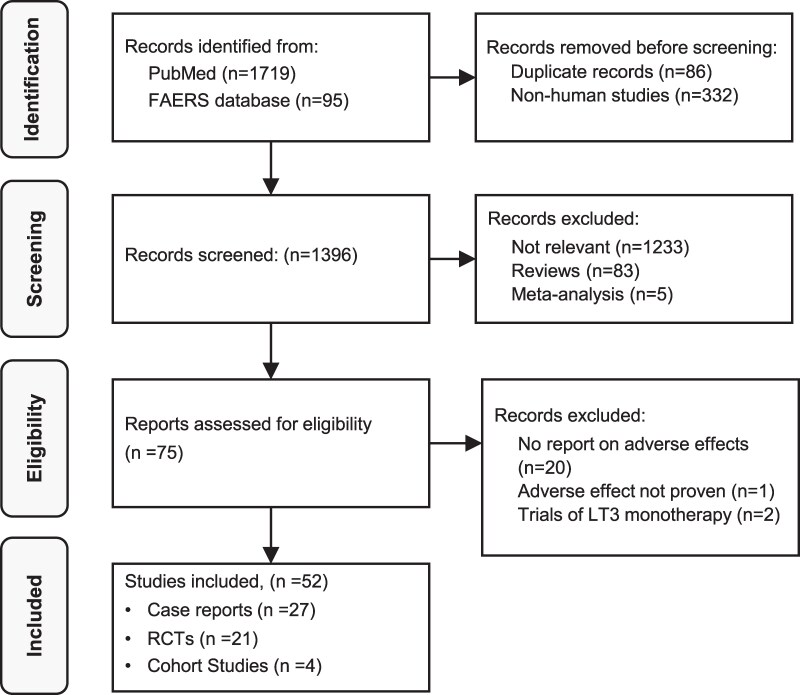
Flow chart for study inclusions.

The original search identified 1814 articles, including published reports in drug monitoring databases. We excluded 418 articles comprising 86 duplicates and 332 articles with nonhuman subjects. A further 1321 articles were excluded after title or abstract screening, and 23 papers were excluded after full text review, resulting in a total number of 52 papers in the review. The selected studies comprised 27 case reports, 4 cohort studies, and 21 RCTs. A study flow chart and reasons for exclusions on full text review are shown in Fig. 1.

### Case Reports

We identified 28 cases of serious AEs linked to LT3 in 25 reports published between 1979 and 2024 (21, 30-53). We excluded 1 report of sagittal sinus thrombosis in a patient on liothyronine and Armour Thyroid due to the presence of other more plausible etiological factors, including a positive screen for prothrombin gene deficiency (32). Of the remaining 27 cases, 59% were female with an age range of 20 to 71 years (Table 1). AEs were mostly due to pharmacy compounding errors (10 cases) or the unlicensed use of LT3 for bodybuilding, weight loss, or fatigue in individuals without hypothyroidism (14 cases). Compounding errors involved the ingestion of LT3 doses that were 10 to 1000 times the intended doses equivalent to the ingestion of LT3 at dose ranges of 1000 to 50 000 mcg daily. Thyrotoxicosis, thyroid storm, or thyrotoxic periodic paralysis were the most frequently reported AEs, and these usually resolved on discontinuing the offending preparation. Two fatalities were reported, 1 in a 29-year-old male without a history of hypothyroidism who had self-administered 100 mcg of LT3 daily for 6 months for weight loss purposes (47) and the other in a 42-year-old woman who ingested an unspecified amount of LT3 that had been procured over the internet presumably for fatigue (21). No AE was reported for patients with hypothyroidism who received supervised treatment with standard LT3 doses under licensed indications (Table 1).

**Table 1.** Case reports of serious adverse effects associated with LT3

First author, year (reference)	Age, sex	Treatment, dose, duration	Adverse outcome
Pharmacy compounding errors			
Sola, 2002 (41)	44, F	LT3 ∼ 50 000 mcg daily, 8 days	Thyroid storm
Shah, 2017 (42)	53, F	LT3 in thyroid extracts, dose NS	Thyrotoxicosis
Khan, 2019 (40)	30, F	LT3 ∼ 30 000 mcg daily, 2 weeks	Thyroid storm
Bains, 2015 (45)	71, F	LT3, dose NS	Myocardial infarction
Bains, 2015 (45)	59, F	LT3, dose NS	Thyrotoxicosis
He, 2020 (48)	20, M	LT3, 10 794 mcg	Thyrotoxicosis
He, 2020 (48)	30, F	LT3, 30 000 mcg daily, 16 days	Thyroid storm
He, 2020 (48)	44, F	LT3, 10× usual dose	Thyrotoxicosis
DL Calzada, 2011 (49)	43, F	LT3, 1300 mcg daily	Thyrotoxicosis
Jha, 2012 (50)	62, F	LT3 in thyroid extracts, dose NS	Thyroid storm
Pharmacy dispensing error			
Manasra, 2023 (31)	53, M	LT3 15 mcg daily, erroneously dispensed instead of MMI	Atrial fibrillation
Unlicensed use for weight loss			
Cheema 2018 (38)	33, M	LT3-containing supplements	Thyrotoxic periodic paralysis
Regina, 2016 (43)	30, F	LT3 diet pills, 625 mcg, 2 weeks	Thyrotoxicosis
Chou, 2009 (46)	23, M	LT3, 64 mcg daily, 4 weeks	Thyrotoxic periodic paralysis
Hartung, 2010 (47)	29, M	LT3, 100 mcg, 6 months	Fatal thyroid storm
Akinyemi, 2011 (51)	35, F	LT3 in diet pills, dose NS	Thyrotoxic periodic paralysis
Panikkath, 2014 (52)	24, M	LT3 in diet pills, dose NS	Thyrotoxic periodic paralysis
Daniels, 2013 (53)	49, F	LT3 in diet pills	Thyrotoxicosis
Unlicensed use for fatigue			
Shaw, 2021 (30)	58, F	LT3, 5-40 mcg daily, 6 weeks	Stress cardiomyopathy
Parimi, 2021 (35)	52, F	LT3, 250 mcg daily, 18 months	Thyrotoxicosis
Bahl, 2024 (21)	42, F	LT3, dose NS	Sudden death
Unlicensed use for bodybuilding			
Miklin, 2023 (33)	31, M	LT3, dose NS	Cardiomyopathy
Warner, 2020 (34)	25, M	LT3, 25 mcg daily, 4 weeks	Persistent tachycardia
Kwak, 2016 (44)	25, M	LT3, 75 mcg daily, 3 weeks	Thyrotoxicosis
Van Bokhorst, 2021 (36)	29, M	LT3, 50-100 mcg daily, 8 years	Thyrotoxic periodic paralysis
Intentional overdose			
Quan, 2016 (39)	50, M	LT3, 180 tablets, dose NS	Thyroid storm
Dahlberg, 1979 (37)	30, F	LT3, 1600 mcg	Thyrotoxicosis

Abbreviations: F, female; LT3, liothyronine; M, male; mcg, microgram; MMI, methimazole; NS, not stated.

### RCTs

We initially reviewed 23 RCTs of LT3 efficacy (54-76). We excluded trials that did not provide information on AEs (72, 74) or did not distinguish AE between the trial arms (71) and trials that only addressed LT3 monotherapy vs LT4 monotherapy (75, 76). Details of RCTs of combination LT3/LT4 vs LT4 monotherapy with AE reports are shown in Table 2 (54-70, 73). Reported AEs mostly included symptoms of hyperthyroidism or hypothyroidism. Only 1 study reported significantly increased AEs in the combination group. This study, conducted by Smith et al in the 1960s (69), administered relatively high LT3 doses of 40 to 60 mcg daily, in combination with LT4 doses of 200 to 300 mcg. No statistically significant differences were noted in any of the other studies between AEs recorded in treatment or control arms, and none of the RCTs reported any sudden deaths (Table 2).

**Table 2.** AEs in RCTs of combination LT3/LT4 vs LT4 monotherapy

First author, year (reference)	RCT design	AEs reported	n	Duration	LT4 dose	LT4/LT3 dose
Appelhof, 2005 (54)	Parallel	No difference	141	15 weeks	LT4 usual dose	LT4/LT3 in 10:1 ratio or 5:1 ratio
Biondi, 2023 (55)	Parallel	No difference	28	1 year	Usual LT4 dose	Usual LT4 dose/LT3 in 17:1 ratio
Brigante, 2024 (56)	Parallel	No difference	141	6 months	Usual LT4 dose	Usual LT4 dose/LT3 in 13-20:1 ratio
Bunevicius, 1999 (73)	Cross-over	No difference	31	5 weeks	Usual LT4 dose	Usual LT4 dose minus 50 mcg/LT3 12.5 mcg
Clyde, 2003 (57)	Parallel	No AEs reported	46	4 months	Usual LT4 dose	50% usual LT4 dose/LT3 7.5 mcg
E-Morreale, 2005 (58)	Cross-over	No difference	28	8 weeks	LT4 100 mcg	LT4 75-87.5 mcg/LT3 5-7.5 mcg
Hoang, 2013 (59)	Cross-over	No difference	70	16 weeks	LT4 75-225 mcg	DTE 43-172 mg
Kaminski, 2016 (60)	Cross-over	No AEs reported	32	8 weeks	LT4 125-150 mcg	LT4 125-150 mcg/LT3 7.5-15 mcg
Krysiak, 2018 (61)	Quasi-randomized	No difference	39	6 months	Usual LT4 dose	50% LT4 dose/LT3 in 5:1 ratio
Nygaard, 2009 (62)	Cross-over	No difference	59	12 weeks	Usual LT4 dose	Usual LT4 dose/LT3 20 mcg
Rodriguez, 2006 (63)	Cross-over	No difference	30	6 weeks	Usual LT4 dose	Usual LT4 dose minus 50 mcg/LT3 10 mcg
Saravanan, 2005 (64)	Parallel	No difference	697	1 year	Usual LT4 dose	Usual LT4 dose minus 50 mcg/LT3 10 mcg
Sawka, 2003 (65)	Parallel	No difference	40	15 weeks	Usual LT4 dose	50% LT4 dose/LT3 12.5 mcg
Shakir, 2021 (66)	Cross-over	No AEs reported	75	22 weeks	LT4 88-250 mcg	LT4 63-175 mcg/LT3 7.5-20 mcg
Siegmund, 2004 (67)	Cross-over	No AEs reported	23	12 weeks	LT4 100-175 mcg	LT4 100-175 mcg/LT3 5-10 mcg
Slawik, 2007 (68)	Cross-over	No difference	29	5 weeks	LT4 1.6 mcg/kg	LT4 1.4 mcg/kg/LT3 0.16 mcg/kg
Smith, 1970 (69)	Cross-over	More AEs in LT3/LT4 arm	87	2 months	LT4 200-300 mcg	LT4 200-300 mcg/LT3 40-60 mcg
Valizadeh, 2009 (70)	Parallel	No AEs reported	71	4 months	LT4 100 mcg	LT4 50 mcg/LT3 6.25 mcg

Abbreviations: AEs, adverse events; DTE, desiccated thyroid extracts; LT3, liothyronine; LT4, levothyroxine; mcg, micrograms; RCT, randomized controlled trial.

**Figure 2.**
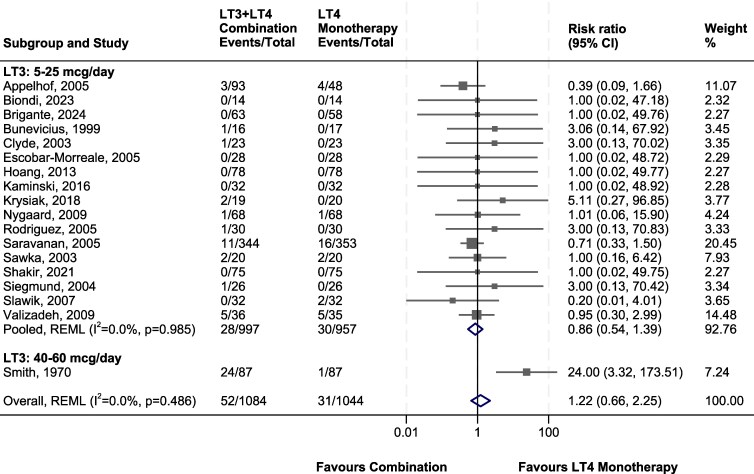
Meta-analysis of withdrawals from randomized controlled trials due to adverse effects. Abbreviation: REML, restricted maximum likelihood.

Of the included studies, 18 trials contained information on the number of trial participants in each study arm who discontinued treatment or withdrew from trials due to AEs (54-70, 73). The numbers of AE withdrawals or treatment discontinuation for trials of combination LT3/LT4 vs LT4 monotherapy were pooled in a meta-analysis and grouped by administered dose (Fig. 2). LT3 was administered in combination with LT4 at average daily doses of 5 to 25 mcg daily with the exception of the high-dose study by Smith et al, which administered 40 to 60 mcg daily in combination with LT4 doses of 200 to 300 mcg and showed increased risk of AEs (69) (Fig. 2). However, despite inclusion of this study in the meta-analysis, there was no overall increased risk of AE withdrawals in the combination vs LT4 monotherapy groups (Fig. 2). The lack of association persisted after excluding trials with 0 AE withdrawals (Supplementary Fig. S1) (29).

### Cohort Studies

**Figure 3.**
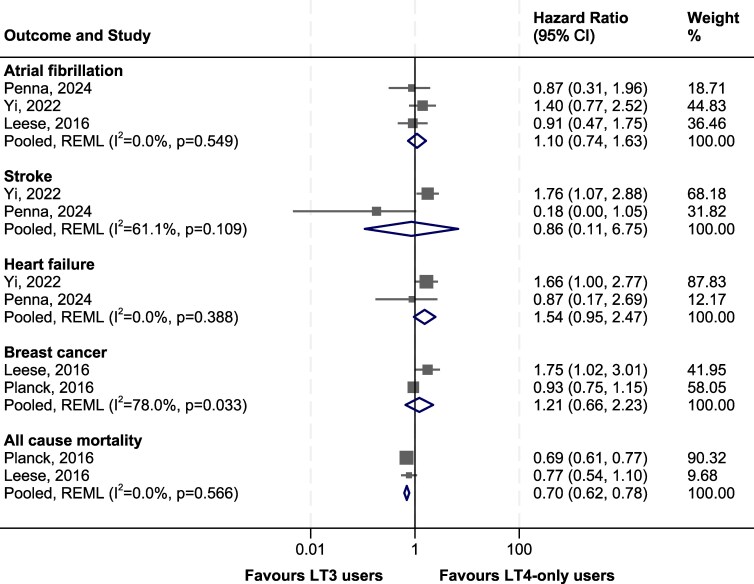
Meta-analysis of cohort studies. Abbreviations: REML, restricted maximum likelihood.

We identified 4 cohort studies, 1 each from Scotland (77), Sweden (78), Korea (79), and the United States (80), that reported morbidity and mortality data in LT3 vs LT4 users (Table 3). Studies were all retrospective observational studies using national or health expenditure datasets with mean follow-up periods ranging from over 90 days to 9 years (77-80). Studies were of moderate to good quality (Supplementary Table S2) (29). Patients were grouped into LT3 users (either alone or in combination with LT4, n = 13 060) vs users of LT4 alone (n = 616 942). One study also included patients on DTE (n = 252) in its LT3 group (80). Cohorts included varying proportions of thyroid cancer patients (Table 3). The study by Leese et al comprised 0.4% thyroid cancer patients in the LT4 group and 22% in the LT3 group (77). The other 2 studies contained <5% of patients with thyroid cancer in either group (78, 80). In contrast, the study by Yi et al comprised higher rates of thyroid cancer patients (40% in the LT3 group and 35% in the LT4 group) (79). Outcomes reported in at least 2 studies are summarized in Fig. 3 and include all-cause mortality (2 studies), atrial fibrillation (3 studies), strokes (2 studies), heart failure (2 studies), and breast cancer (2 studies). Event rates were summarized as hazard ratios (HRs) and 95% CIs.

**Table 3.** Cohort studies on mortality and cardiovascular events for levothyroxine vs liothyronine users

First author, year, country (reference)	Study design	Mean age, sex	Variables adjusted for	Mean follow-up	Numbers, exposures, indications	Outcomes
Leese, 2016, Scotland (77)	Retrospective cohort population study of national registries	LT3 47 yrs, 84% female LT4 60 yrs, 81% female	Age, sex, thyroid cancer, past hyperthyroidism, baseline TSH	9.3 yrs	LT3, 400 (22% thyroid cancer), LT4 only, 34 000 (0.3% thyroid cancer)	Mortality, cardiovascular disease, atrial fibrillation, fractures, breast cancer, and mental disorders
Planck, 2021, Sweden (78)	Retrospective cohort population study of national registries	LT3 46 yrs, 91% female LT4 59 yrs, 82% female	Age, dose, sex, thyroid cancer and other cancers, antithyroid drug and sex hormone use	8.1 yrs	LT3, 11 147 (1.2% thyroid cancer), LT4 only, 573 928 (0.8% thyroid cancer)	Mortality, breast cancer, any cancer
Yi, 2022, Korea (79)	Retrospective, cohort study of hospital databases	Median age 40-50 yrs LT3 84% female LT4 82% female	Propensity scores matched for age, sex, and comorbidity	Over 90 days	LT3, 1434 (40% thyroid cancer), LT4 only, 3908 (35% thyroid cancer)	Atrial fibrillation, heart failure, ischaemic heart disease, stroke, cancer, anxiety and mood disorders, osteoporosis
Penna, 2024, USA (80)	Retrospective cohort study of medical expenditure database	LT3 61 yrs, 97% female LT4 61 yrs, 76% female, DTE 56 yrs, 92% female	Age, sex, race, ethnicity, family income, survey year, comorbidities	Median 6 yrs (LT4), 3 yrs (LT3/LT4 and DTE)	LT4, 5106, (3.6% thyroid cancer), DTE, 252 (3.0% thyroid cancer), LT3, 79 (1.3% thyroid cancer)	Atrial fibrillation, heart failure, myocardial infarction, stroke, osteoporosis, fracture

Abbreviations: DTE, desiccated thyroid extracts; LT3, liothyronine users; LT4, levothyroxine only users.

In the meta-analysis, LT3 use was not associated with a significantly increased risk of any of the reported outcomes (Fig. 3). There was a nonsignificant increased risk of heart failure with combination therapy (HR 1.54, 95% CI 0.95, 2.47), driven by the large Korean study by Yi et al (79). Further subgroup analyses of this cohort showed that increased heart failure risk was only seen in patients with a history of thyroid cancer, raising the possibility of unaccounted risk factors such as targeted thyroid hormone suppression in this cohort. In contrast, pooled analysis of the 2 studies that reported on all-cause mortality showed a reduction in mortality (HR 0.70, 95% CI 0.62-0.78) in LT3 users, driven by the large Swedish study by Planck et al (Fig. 3).

### Yellow Card Reports

Data from Yellow Card reports were generated for LT3 and LT4 from 1967 to 2024. In that period, 3226 adverse reports were linked to LT4, comprising 14 663 reactions, while 292 reports and 1687 reactions were linked to LT3. The breakdown of reactions according to the Medical Dictionary for Regulatory Activities System Organ Class is presented in Supplementary Fig. S2 (29), which summarizes reactions reported in each system as a percentage of the total reactions for each drug. Both drugs showed similar AE profiles, with most reactions being general disorder events, nervous system, or psychiatric illness disorders (Supplementary Fig. S2) (29). For LT3, 22% of all reactions were general disorder events compared to 17% of LT4 reactions. In contrast, gastric and skin reactions accounted for 11% and 10% of LT4 reactions compared to 7% and 7% of LT3 reactions, respectively. The breakdown of reports according to severity is shown in Supplementary Fig. S3 (29). Similar rates of serious and nonserious AEs were reported for LT4 and LT3, with 1 death reported in association with LT4 and no reported deaths for LT3.

### The FAERS Database

A disproportionality analysis using the FAERS database did not show any signal for LT3 either for serious AEs or deaths (Table 4). These results were consistent for the ROR, proportional reporting ratio, and ICs, which are drug safety signal measures derived from 2 × 2 contingency tables in disproportionality analyses (Supplementary Table S1) (29). Increased signal in the ROR and IC was seen for LT4 for serious AEs but not for deaths (Table 4).

**Table 4.** Disproportionality analysis

AEs	Cases, n	Non-cases, n	Disproportionality measures		
	Target drug/other drugs (A/C)	Target drug/other drugs (B/D)	ROR (95% CI)	PRR (95% CI)	IC (95% CI)
LT3, serious AEs	1212/10 974 443	2085/9 840 513	0.52 (0.49, 0.56)	0.70 (0.25, 1.98)	−0.61 (−0.52, −0.45)
LT4, serious AEs	44 545/10 931 110	27 667/9 814 930	1.45 (1.42, 1.47)*^a^*	1.17 (0.42, 3.30)	0.21 (0.23, 0.24)^b^
LT3, deaths	56/1 777 557	3241/19 037 400	0.19 (0.14, 0.24)	0.20 (0.17, 0.24)	−2.75 (−2.32, −2.00)
LT4, deaths	4917/1 772 696	67 295/18 973 344	0.78 (0.76, 0.81)	0.80 (0.67, 0.94)	−0.37 (−0.33, −0.29)

The table shows ROR, PRR, and IC for LT3 and LT4 using the US Food and Drug Administration Adverse Reporting System database.

Abbreviations: A/C, adverse effect of interest reported with target drug/adverse effect of interest reported with other drugs; AE, adverse event; B/D, other adverse effects reported with target drug/other adverse effects reported with other drugs; CI, confidence interval; IC, information components; LT3, liothyronine; LT4, levothyroxine; PRR, proportional reporting ratio; ROR, reporting odds ratio.

^*a*^Significant signals.

## Discussion

We conducted a systematic review and meta-analysis on the safety of LT3 using a variety of sources comprising case reports, cohort studies, RCTs, and pharmacovigilance databases. Our findings suggest that LT3, when used within medically recommended doses and under appropriate supervision, is not associated with an increased risk of serious AEs, cardiovascular events, or death. Case reports revealed that serious AEs were almost exclusively linked to supratherapeutic LT3 exposure, either through compounding errors, unregulated online procurement, or misuse for weight loss and bodybuilding. These cases often involved doses 10 to 1000 times above therapeutic levels. Importantly, no adverse outcomes were reported when LT3 was prescribed and monitored in line with clinical guidelines.

A meta-analysis of RCTs showed no increase in AE-related treatment discontinuation in combination LT4/LT3 therapy compared to LT4 monotherapy, even when trials using higher LT3 doses were included. Similarly, cohort studies involving over 13 000 LT3 users found no excess risk of atrial fibrillation, heart failure, or stroke. Notably, pooled mortality data from 2 large studies showed a statistically significant reduction in all-cause mortality among LT3 users compared with LT4 users. Pharmacovigilance data provided further reassurance on LT3 safety. Yellow Card reports from the United Kingdom and FAERS data from the United States revealed similar AE profiles for LT3 and LT4. Disproportionality analysis did not identify a safety signal for LT3 but did show an increased reporting signal for serious AEs with LT4, albeit not for mortality.

Ours is the first comprehensive systematic review of its kind to collate evidence from multiple sources on the safety of LT3. Also, we have presented the first meta-analysis of cohort studies on cardiovascular and mortality outcomes associated with LT3 use. AE risks in RCTs were only specifically addressed in a few previous systematic reviews (11, 12). One study by Millan-Alanis et al, which described AEs in RCTs without a formal meta-analysis, concluded that overall incidence of AEs in the 2 arms did not differ (11). A meta-analysis of RCTs by Grozinsky-Glasberg et al showed similar results to ours with no significant increase in AE risk for LT3 compared to LT4 (12). However, these meta-analyses were limited to trials that met criteria for efficacy analysis. In contrast, our study included more RCTs regardless of the primary study outcome, as the key interest of our analysis was drug safety. Our findings are also broadly consistent with a recent narrative review that reported a low number of spontaneous AEs for a branded LT3–LT4 product based on market safety information in the Merck global pharmacovigilance database (20). In contrast to this study, however, our study was systematic, evaluated all available LT3 products in independent national datasets, and employed validated pharmacovigilance and meta-analyses methods to establish short- and long-term safety outcomes.

Our findings have implications for the treatment of hypothyroidism. Although LT4 remains the recommended treatment in hypothyroidism, a proportion of patients appear best served with combination LT3/LT4 therapy. A limitation to the use of combination therapy is the paucity of safety data. Earlier studies in the 1960s suggested that LT3 was associated with an excess risk of thyrotoxicosis and its cardiovascular complications (69). However, as our review shows, such reported risks are inconsistent with data from more recent studies and are likely to have been a function of excessive LT3 dosing. A large cohort study from Korea reported an increased risk of heart failure and strokes among LT3 users compared to LT4 users (79). This study, however, comprised a high proportion of patients who had been treated for thyroid cancer and would conceivably have been on TSH-suppressive treatment doses. Furthermore, this effect was lost when pooled in a meta-analysis with another study from the United States (80). Interestingly, a pooled analysis of 2 studies from the United Kingdom (77) and Sweden (78) that addressed all-cause mortality showed reduced mortality risk for LT3 compared to LT4. However, this finding should be interpreted with caution as it may point to unexplored factors in the selection of patients for LT3 treatment. Nonetheless, our data show that LT3 is at least as safe as LT4 and should inform treatment choices in patients with hypothyroidism.

Our study has several limitations. The drug databases rely on reports submitted by patients, pharmacists, healthcare workers, and medical professionals, and underreporting is a well-known limitation of such datasets. In addition, the nature of symptoms associated with LT3 treatment may be subjective, with a lack of objective proof linking LT3 to AEs. Furthermore, LT3 reporting may be subject to notoriety bias due to publicized safety concerns. Lastly, the disproportionality analyses are only measures of drug safety signals and cannot be considered indicators of association or causality (27). The RCTs trials also suffer from limitations in terms of inadequate power for AE analysis, short follow-up durations, and possible bias by association with underlying diagnoses and cardiovascular risk. Furthermore, several RCTs could not be included in our meta-analysis as these studies did not report on the incidence of AEs. Lastly, the cohort studies were heterogeneous in study design, patient characteristics, and outcomes, and due to the small number of published studies, the meta-analysis was limited to 2 to 3 studies per individual outcome.

These limitations therefore underscore the need for further studies to establish the safety of LT3. Further large cohort studies will be particularly insightful as they will reflect practice in real-world cohorts with longer follow-up durations, which is challenging to capture within the limited timeframe of controlled trials. Such studies should ideally include an evaluation of treatment dose and duration as well as thyroid hormone status to better understand the role of treatment dose and TSH suppression in mitigating outcome risks. Assessments of exposure to other alternative therapies apart from combination therapy, including LT3 monotherapy and DTEs, should also be undertaken and will provide valuable insights into these increasingly used treatment approaches. Future trials on combination therapy addressing the limitations of existing trials are likely to be undertaken given that the evidence surrounding the benefits of combination therapy remains unproven (18). It is important that future RCTs report specifically on AEs and treatment withdrawals according to established clinical trials procedures.

In conclusion, our systematic review addresses a critical gap in the growing need for safety assurance around the use of LT3, given the strong patient preference, hesitancy of clinicians to prescribe LT3, and potential for abuse by patients self-medicating from unregulated sources. Our report highlights the real risks of LT3 misuse outside medical supervision, together with the surprising frequency of pharmacy compounding errors. On the other hand, when used and monitored appropriately by registered physicians, LT3 is safe and well tolerated and is not associated with increased AE risks, emphasizing that the frequency of LT3-associated harm appears to reflect misuse rather than pharmacologic toxicity. Thus, in view of the analysis conducted here, where doses are not excessive, the occurrence of a sudden death in an LT3 user may be just as likely to be coincidental as in an LT4 user, especially when considering similar risks reported with LT4. Thus, while clinicians should remain vigilant regarding unregulated LT3 use and compounding practices, these risks should not preclude its use in medically supervised settings.

## Data Availability

Original data was generated for this study from data in the published literature. These data are included in this article and in the data repository listed in the References.

## References

[1] Murray GR . Note on the treatment of myxoedema by hypodermic injections of an extract of the thyroid gland of a sheep. Br Med J. 1891;2(1606):796‐797. 10.1136/bmj.2.1606.796 PMC2273741 20753415

[2] Taylor PN, Albrecht D, Scholz A, et al Global epidemiology of hyperthyroidism and hypothyroidism. Nat Rev Endocrinol. 2018;14(5):301‐316. 29569622 10.1038/nrendo.2018.18

[3] Taylor PN, Medici MM, Hubalewska-Dydejczyk A, Boelaert K. Hypothyroidism. Lancet. 2024;404(10460):1347‐1364. 39368843 10.1016/S0140-6736(24)01614-3

[4] Chaker L, Bianco AC, Jonklaas J, Peeters RP. Hypothyroidism. Lancet. 2017;390(10101):1550‐1562. 28336049 10.1016/S0140-6736(17)30703-1 PMC6619426

[5] Okosieme OE . Thyroid hormone replacement: current status and challenges. Expert Opin Pharmacother. 2011;12(15):2315‐2328. 21762038 10.1517/14656566.2011.600307

[6] Salvatore D, Porcelli T, Ettleson MD, Bianco AC. The relevance of T(3) in the management of hypothyroidism. Lancet Diabetes Endocrinol. 2022;10(5):366‐372. 35240052 10.1016/S2213-8587(22)00004-3 PMC9987447

[7] Taylor PN, Razvi S, Muller I, et al Liothyronine cost and prescriptions in England. Lancet Diabetes Endocrinol. 2019;7(1):11‐12. 30577888 10.1016/S2213-8587(18)30334-6

[8] Peterson SJ, Cappola AR, Castro MR, et al An online survey of hypothyroid patients demonstrates prominent dissatisfaction. Thyroid. 2018;28(6):707‐721. 29620972 10.1089/thy.2017.0681 PMC6916129

[9] Saravanan P, Chau WF, Roberts N, Vedhara K, Greenwood R, Dayan CM. Psychological well-being in patients on ‘adequate’ doses of l-thyroxine: results of a large, controlled community-based questionnaire study. Clin Endocrinol (Oxf). 2002;57(5):577‐585. 12390330 10.1046/j.1365-2265.2002.01654.x

[10] Okosieme O, Gilbert J, Abraham P, et al Management of primary hypothyroidism: statement by the British Thyroid Association Executive Committee. Clin Endocrinol (Oxf). 2016;84(6):799‐808. 26010808 10.1111/cen.12824

[11] Millan-Alanis JM, González-González JG, Flores-Rodríguez A, et al Benefits and harms of levothyroxine/L-triiodothyronine versus levothyroxine monotherapy for adult patients with hypothyroidism. Systematic review and meta-analysis. Thyroid. 2021;31(11):1613‐1625. 34340589 10.1089/thy.2021.0270 PMC8917901

[12] Grozinsky-Glasberg S, Fraser A, Nahshoni E, Weizman A, Leibovici L. Thyroxine–triiodothyronine combination therapy versus thyroxine monotherapy for clinical hypothyroidism: meta-analysis of randomized controlled trials. J Clin Endocrinol Metab. 2006;91(7):2592‐2599. 16670166 10.1210/jc.2006-0448

[13] Ma C, Xie J, Huang X, et al Thyroxine alone or thyroxine plus triiodothyronine replacement therapy for hypothyroidism. Nucl Med Commun. 2009;30(8):586‐593. 19491714 10.1097/MNM.0b013e32832c79e0

[14] de Lima Beltrão FE, Carvalhal G, de Almeida Beltrão DC, et al Treatment preferences in patients with hypothyroidism. J Clin Endocrinol Metab. 2025;110(3):887‐900. 39290156 10.1210/clinem/dgae651 PMC11834714

[15] Jonklaas J, Bianco AC, Bauer AJ, et al Guidelines for the treatment of hypothyroidism: prepared by the American Thyroid Association task force on thyroid hormone replacement. Thyroid. 2014;24(12):1670‐1751. 25266247 10.1089/thy.2014.0028 PMC4267409

[16] Wiersinga WM, Duntas L, Fadeyev V, Nygaard B, Vanderpump MP. 2012 ETA guidelines: the use of L-T4 + L-T3 in the treatment of hypothyroidism. Eur Thyroid J. 2012;1(2):55‐71. 24782999 10.1159/000339444 PMC3821467

[17] Ahluwalia R, Baldeweg SE, Boelaert K, et al Use of liothyronine (T3) in hypothyroidism: Joint British Thyroid Association/Society for endocrinology consensus statement. Clin Endocrinol (Oxf). 2023;99(2):206‐216. 37272400 10.1111/cen.14935

[18] Jonklaas J, Bianco AC, Cappola AR, et al Evidence-based use of levothyroxine/liothyronine combinations in treating hypothyroidism: a consensus document. Eur Thyroid J. 2021;10(1):10‐38. 33777817 10.1159/000512970 PMC7983670

[19] Idrees T, Palmer S, Maciel RMB, Bianco AC. Liothyronine and desiccated thyroid extract in the treatment of hypothyroidism. Thyroid. 2020;30(10):1399‐1413. 32279609 10.1089/thy.2020.0153 PMC7640752

[20] Gottwald-Hostalek U, Tayrouz Y. A review of the safety of triiodothyronine in combination with levothyroxine for the management of hypothyroidism. Curr Med Res Opin. 2024;40(12):2109‐2116. 39625345 10.1080/03007995.2024.2435460

[21] Bahl S, Taylor P, Okosieme O, et al Liothyronine and a sudden unexplained death: cause or coincidence? Endocr Abstr. 2024;101:PS1-07-02.

[22] FDA . Data from: FDA Adverse Event Reporting System (FAERS) Public Dashboard. https://www.fda.gov/drugs/questions-and-answers-fdas-adverse-event-reporting-system-faers/fda-adverse-event-reporting-system-faers-public-dashboard

[23] MHRA . Data from: MHRA Yellow Card Scheme iDAPs. 2025. https://info.mhra.gov.uk/drug-analysis-profiles/dap.html?drug=./UK_EXTERNAL/NONCOMBINED/UK_NON_000981416963.zip&agency=MHRA

[24] MedDRA . Data from: Introductory Guide. MedDRA. Version 27.0. 2024. https://alt.meddra.org/files_acrobat/intguide_27_0_English.pdf

[25] Wells GA, Shea B, O’Connell D, et al The Newcastle-Ottawa Scale (NOS) for assessing the quality of nonrandomised studies in meta-analyses. http://www.evidencebasedpublichealth.de/download/Newcastle_Ottawa_Scale_Pope_Bruce.pdf.

[26] Sweeting MJ, Sutton AJ, Lambert PC. What to add to nothing? Use and avoidance of continuity corrections in meta-analysis of sparse data. Stat Med. 2004;23(9):1351‐1375. 15116347 10.1002/sim.1761

[27] Cutroneo PM, Sartori D, Tuccori M, et al Conducting and interpreting disproportionality analyses derived from spontaneous reporting systems. Front Drug Saf Regul. 2024;3:1323057. 40980108 10.3389/fdsfr.2023.1323057 PMC12443087

[28] Norén GN, Hopstadius J, Bate A. Shrinkage observed-to-expected ratios for robust and transparent large-scale pattern discovery. Stat Methods Med Res. 2013;22(1):57‐69. 21705438 10.1177/0962280211403604 PMC6331976

[29] Bahl S, Taylor PN, Premawardhana LD, et al Supplementary data for Risk of death and adverse effects in patients on Liothyronine: a multi-sourcesystematic review and meta-analysis. 2025. 10.6084/m9.figshare.29402231.v1 PMC12527464 40795305

[30] Shaw KE, Jamrozy A. Stress cardiomyopathy due to exogenous thyrotoxicosis from T3 supplementation. J Endocr Soc. 2021;5(Supplement_1):A955.

[31] Manasra A, Shah T, Raj SQ, Pollak SR, T P. Amiodarone thyrotoxicosis: a case of pharmacy error obscured by amiodarone suppression of symptoms of highly elevated thyroxine concentration. CJC Open. 2023;5(2):173‐176. 36880080 10.1016/j.cjco.2022.11.019 PMC9984889

[32] Shin TH, Smith P, Goulden P. SAT-519 armour thyroid and liothyronine combination potentiated thrombosis. J Endocr Soc. 2020;4(Supplement_1):2020.

[33] Miklin D, Marecki G, Weintraub SF, Kuvin JT. Bodybuilder's cardiomyopathy: a unique case of hormone induced heart failure. J Am Coll Cardiol. 2023;81(8):2973‐2973.

[34] Warner BE, Woodrow CJ, Pal A. Delayed diagnosis of T3 supplementation in a bodybuilder presenting with tachycardia and features of sepsis. BMJ Case Rep. 2020;13(1):e232867. 10.1136/bcr-2019-232867 PMC7035845 31937628

[35] Parimi J, Gundluru R, Kurukulasuriya R, Naha S. Abstract #1003897: physician induced psychosis. Endocr Pract. 2021;27(6):S173‐S174.

[36] van Bokhorst QNE, Krul-Poel YHM, Smit DL, de Ronde W. A 29-year-old bodybuilder with liothyronine-induced thyrotoxic hypokalaemic periodic paralysis. Eur J Case Rep Intern Med. 2019;8(3):002362. 10.12890/2021_002362 PMC8046276 33869098

[37] Dahlberg PA, Karlsson FA, Wide L. Triiodothyronine intoxication. Lancet. 1979;314(8144):700. 10.1016/s0140-6736(79)92105-6 90795

[38] Cheema MA, Zain MA, Cheema K, Ullah W. Thyroxine-induced periodic paralysis: a rare complication of nutritional supplements. BMJ Case Rep. 2018;11(1):e227946. 10.1136/bcr-2018-227946 PMC6301584 30567254

[39] Quan D, Guynes A. 1688: acute *Thyrotoxicosis factitia*: thyrotoxicosis secondary to acute overdose of exogenous t3 and t4. Crit Care Med. 2016;44(12):497.

[40] Khan W, Van Der Gugten G, Holmes DT. Thyrotoxicosis due to 1000-fold error in compounded liothyronine: a case elucidated by mass spectrometry. Clin Mass Spectrom. 2019;11:8‐11. 34841067 10.1016/j.clinms.2018.11.003 PMC8620522

[41] Solá E, Gómez-Balaguer M, Morillas C, et al Massive triiodothyronine intoxication: efficacy of hemoperfusion? Thyroid. 2002;12(7):637‐640. 12193311 10.1089/105072502320288528

[42] Shah KK, Mbughuni MM, Burgstaler EA, Block DR, Winters JL. Iatrogenic thyrotoxicosis and the role of therapeutic plasma exchange. J Clin Apher. 2017;32(6):579‐583. 28319287 10.1002/jca.21536

[43] Regina A, Majlesi N. Notes from the field: thyrotoxicosis after consumption of dietary supplements purchased through the Internet–Staten Island, New York, 2015. MMWR Morb Mortal Wkly Rep. 2016;65(13):353‐354. 27054849 10.15585/mmwr.mm6513a4

[44] Kwak T, Al Zoubi M, Bhavith A, Rueda Rios C, Kumar S. Acute myocarditis in bodybuilder from coxsackievirus and thyrotoxicosis. J Cardiol Cases. 2016;14(4):123‐126. 30524566 10.1016/j.jccase.2016.06.005 PMC6262103

[45] Bains A, Brosseau AJ, Harrison D. Iatrogenic thyrotoxicosis secondary to compounded liothyronine. Can J Hosp Pharm. 2015;68(1):57‐59. 25762821 10.4212/cjhp.v68i1.1426 PMC4350501

[46] Chou HK, Tsao YT, Lin SH. An unusual cause of thyrotoxic periodic paralysis: triiodothyronine-containing weight reducing agents. Am J Med Sci. 2009;337(1):71‐73. 19002009 10.1097/01.MAJ.0000310783.66897.b6

[47] Hartung B, Schott M, Daldrup T, Ritz-Timme S. Lethal thyroid storm after uncontrolled intake of liothyronine in order to lose weight. Int J Legal Med. 2010;124(6):637‐640. 20145940 10.1007/s00414-010-0423-y

[48] He ZH, Li Y, Trivedi N, Gill S, Hennessey JV. Thyrotoxicosis after massive triiodothyronine (LT3) overdose: a coast-to-coast case series and review. Drugs Context. 2020:9:2019-8-4. 10.7573/dic.2019-8-4 PMC7048132 32158485

[49] De La Calzada-Jeanlouie M, Greller H, Su M, Chan G. A case of thyrotoxicosis due to a compounding error. Clin Toxicol. 2011;49(6):553‐553.

[50] Jha S, Waghdhare S, Reddi R, Bhattacharya P. Thyroid storm due to inappropriate administration of a compounded thyroid hormone preparation successfully treated with plasmapheresis. Thyroid. 2012;22(12):1283‐1286. 23067331 10.1089/thy.2011.0353

[51] Akinyemi E, Bercovici S, Niranjan S, Paul N, Hemavathy B. Thyrotoxic hypokalemic periodic paralysis due to dietary weight-loss supplement. Am J Ther. 2011;18(3):e81‐e83. 20068442 10.1097/MJT.0b013e3181c960a9

[52] Panikkath R, Nugent K. I lost weight, but I became weak and cannot walk—a case of nutraceutical (T3)-induced thyrotoxic periodic paralysis. Am J Ther. 2014;21(6):e211‐e214. 23567793 10.1097/MJT.0b013e318288a460

[53] Daniels GH, Sluss P. Pure T3-thyrotoxicosis from a Mexican weight loss supplement. Endocr Pract. 2013;19(3):558–5560. 23425657 10.4158/EP12388.LT

[54] Appelhof BC, Fliers E, Wekking EM, et al Combined therapy with levothyroxine and liothyronine in two ratios, compared with levothyroxine monotherapy in primary hypothyroidism: a double-blind, randomized, controlled clinical trial. J Clin Endocrinol Metab. 2005;90(5):2666‐2674. 15705921 10.1210/jc.2004-2111

[55] Biondi B, Pucci M, Pontieri G, Formisano P, Esposito R. Preliminary results of a double-blind randomized controlled trial evaluating the cardiometabolic effects of levothyroxine and liothyronine compared to levothyroxine with placebo in athyreotic low-risk thyroid cancer patients. Thyroid. 2023;33(12):1402‐1413. 37725587 10.1089/thy.2023.0135

[56] Brigante G, Santi D, Boselli G, et al Randomized double-blind placebo-controlled trial on levothyroxine and liothyronine combination therapy in totally thyroidectomized subjects: the LEVOLIO study. Eur J Endocrinol. 2024;190(1):12‐22. 38124252 10.1093/ejendo/lvad172

[57] Clyde PW, Harari AE, Getka EJ, Shakir KM. Combined levothyroxine plus liothyronine compared with levothyroxine alone in primary hypothyroidism: a randomized controlled trial. JAMA. 2003;290(22):2952‐2958. 14665656 10.1001/jama.290.22.2952

[58] Escobar-Morreale HF, Botella-Carretero JI, Gomez-Bueno M, Galan JM, Barrios V, Sancho J. Thyroid hormone replacement therapy in primary hypothyroidism: a randomized trial comparing L-thyroxine plus liothyronine with L-thyroxine alone. Ann Intern Med. 2005;142(6):412‐424. 15767619 10.7326/0003-4819-142-6-200503150-00007

[59] Hoang TD, Olsen CH, Mai VQ, Clyde PW, Shakir MK. Desiccated thyroid extract compared with levothyroxine in the treatment of hypothyroidism: a randomized, double-blind, crossover study. J Clin Endocrinol Metab. 2013;98(5):1982‐1990. 23539727 10.1210/jc.2012-4107

[60] Kaminski J, Miasaki FY, Paz-Filho G, Graf H, Carvalho GA. Treatment of hypothyroidism with levothyroxine plus liothyronine: a randomized, double-blind, crossover study. Arch Endocrinol Metab. 2016;60(6):562‐572. 27982198 10.1590/2359-3997000000192 PMC10522160

[61] Krysiak R, Szkróbka W, Okopień B. Sexual function and depressive symptoms in young women with hypothyroidism receiving levothyroxine/liothyronine combination therapy: a pilot study. Curr Med Res Opin. 2018;34(9):1579‐1586. 29508635 10.1080/03007995.2018.1448771

[62] Nygaard B, Jensen EW, Kvetny J, Jarlov A, Faber J. Effect of combination therapy with thyroxine (T4) and 3,5,3′-triiodothyronine versus T4 monotherapy in patients with hypothyroidism, a double-blind, randomised cross-over study. Eur J Endocrinol. 2009;161(6):895‐902. 19666698 10.1530/EJE-09-0542

[63] Rodriguez T, Lavis VR, Meininger JC, Kapadia AS, Stafford LF. Substitution of liothyronine at a 1:5 ratio for a portion of levothyroxine: effect on fatigue, symptoms of depression, and working memory versus treatment with levothyroxine alone. Endocr Pract. 2005;11(4):223‐233. 16006298 10.4158/EP.11.4.223 PMC1455482

[64] Saravanan P, Simmons DJ, Greenwood R, Peters TJ, Dayan CM. Partial substitution of thyroxine (T4) with tri-iodothyronine in patients on T4 replacement therapy: results of a large community-based randomized controlled trial. J Clin Endocrinol Metab. 2005;90(2):805‐812. 15585551 10.1210/jc.2004-1672

[65] Sawka AM, Gerstein HC, Marriott MJ, MacQueen GM, Joffe RT. Does a combination regimen of thyroxine (T4) and 3,5,3′-triiodothyronine improve depressive symptoms better than T4 alone in patients with hypothyroidism? Results of a double-blind, randomized, controlled trial. J Clin Endocrinol Metab. 2003;88(10):4551‐4555. 14557420 10.1210/jc.2003-030139

[66] Shakir MKM, Brooks DI, McAninch EA, et al Comparative effectiveness of levothyroxine, desiccated thyroid extract, and levothyroxine + liothyronine in hypothyroidism. J Clin Endocrinol Metab. 2021;106(11):e4400‐e4413. 34185829 10.1210/clinem/dgab478 PMC8530721

[67] Siegmund W, Spieker K, Weike AI, et al Replacement therapy with levothyroxine plus triiodothyronine (bioavailable molar ratio 14:1) is not superior to thyroxine alone to improve well-being and cognitive performance in hypothyroidism. Clin Endocrinol (Oxf). 2004;60(6):750‐757. 15163340 10.1111/j.1365-2265.2004.02050.x

[68] Slawik M, Klawitter B, Meiser E, et al Thyroid hormone replacement for central hypothyroidism: a randomized controlled trial comparing two doses of thyroxine (T4) with a combination of T4 and triiodothyronine. J Clin Endocrinol Metab. 2007;92(11):4115‐4122. 17711927 10.1210/jc.2007-0297

[69] Smith RN, Taylor SA, Massey JC. Controlled clinical trial of combined triiodothyronine and thyroxine in the treatment of hypothyroidism. Br Med J. 1970;4(5728):145‐148. 4097650 10.1136/bmj.4.5728.145 PMC1819870

[70] Valizadeh M, Seyyed-Majidi MR, Hajibeigloo H, Momtazi S, Musavinasab N, Hayatbakhsh MR. Efficacy of combined levothyroxine and liothyronine as compared with levothyroxine monotherapy in primary hypothyroidism: a randomized controlled trial. Endocr Res. 2009;34(3):80‐89. 19701833 10.1080/07435800903156340

[71] Walsh JP, Shiels L, Lim EM, et al Combined thyroxine/liothyronine treatment does not improve well-being, quality of life, or cognitive function compared to thyroxine alone: a randomized controlled trial in patients with primary hypothyroidism. J Clin Endocrinol Metab. 2003;88(10):4543‐4550. 14557419 10.1210/jc.2003-030249

[72] Bunevicius R, Jakuboniene N, Jurkevicius R, Cernicat J, Lasas L, Prange AJ. Thyroxine vs thyroxine plus triiodothyronine in treatment of hypothyroidism after thyroidectomy for Graves' disease. Endocrine. 2002;18(2):129‐134. 12374459 10.1385/ENDO:18:2:129

[73] Bunevicius R, Kazanavicius G, Zalinkevicius R, Prange AJ. Effects of thyroxine as compared with thyroxine plus triiodothyronine in patients with hypothyroidism. N Engl J Med. 1999;340(6):424‐429. 9971866 10.1056/NEJM199902113400603

[74] Fadeyev VV, Morgunova TB, Melnichenko GA, Dedov II. Combined therapy with L-thyroxine and L-triiodothyronine compared to L-thyroxine alone in the treatment of primary hypothyroidism. Hormones (Athens). 2010;9(3):245‐252. 20688622 10.14310/horm.2002.1274

[75] Celi FS, Zemskova M, Linderman JD, et al Metabolic effects of liothyronine therapy in hypothyroidism: a randomized, double-blind, crossover trial of liothyronine versus levothyroxine. J Clin Endocrinol Metab. 2011;96(11):3466‐3474. 21865366 10.1210/jc.2011-1329 PMC3205882

[76] Bjerkreim BA, Hammerstad SS, Gulseth HL, et al Effect of liothyronine treatment on quality of life in female hypothyroid patients with residual symptoms on levothyroxine therapy: a randomized crossover study. Front Endocrinol (Lausanne). 2022;13:816566. 35273566 10.3389/fendo.2022.816566 PMC8902821

[77] Leese GP, Soto-Pedre E, Donnelly LA. Liothyronine use in a 17 year observational population-based study—the tears study. Clin Endocrinol (Oxf). 2016;85(6):918‐925. 26940864 10.1111/cen.13052

[78] Planck T, Hedberg F, Calissendorff J, Nilsson A. Liothyronine use in hypothyroidism and its effects on cancer and mortality. Thyroid. 2021;31(5):732‐739. 33040688 10.1089/thy.2020.0388

[79] Yi W, Kim BH, Kim M, et al Heart failure and stroke risks in users of liothyronine with or without levothyroxine compared with levothyroxine alone: a propensity score-matched analysis. Thyroid. 2022;32(7):764‐771. 35570696 10.1089/thy.2021.0634

[80] Penna GC, Bianco AC, Ettleson MD. A cross-sectional analysis of cardiovascular and bone health care utilization during treatment with thyroid hormone. J Clin Endocrinol Metab. 2024;109(3):e1143‐e1150. 37878964 10.1210/clinem/dgad629 PMC10876406

